# An RGB image dataset for seed germination prediction and vigor detection - maize

**DOI:** 10.3389/fpls.2024.1341335

**Published:** 2024-02-21

**Authors:** Chengcheng Chen, Muyao Bai, Tairan Wang, Weijia Zhang, Helong Yu, Tiantian Pang, Jiehong Wu, Zhaokui Li, Xianchang Wang

**Affiliations:** ^1^ School of Computer Science, Shenyang Aerospace University, Shenyang, China; ^2^ College of Information Technology, Jilin Agricultural University, Changchun, China; ^3^ College of Computer Science and Technology, Jilin University, Changchun, China; ^4^ Chengdu Kestrel Artificial Intelligence Institute, Chengdu, China

**Keywords:** RGB image dataset, seed germination prediction, seed vigor detection, maize seed, agriculture, breeding

## 1 Introduction

The greatest food crop in the world, maize, is crucial to ensuring national food security and an efficient supply of agricultural goods (Collins et al., 2018; Hall et al., 2021; Song et al., 2022). The first step in increasing corn yields is to pick high-quality cultivars (Xanthopoulos, 2020; Tu et al., 2022). Seed vigor is an essential test item in the protocols for inspecting the quality of seeds due to it could accurately measure and predict the quality of seed development in the field as well as the potential germination rate, seedling emergence rate, seedling growth potential, plant resistance, and production potential. It is a key indicator for assessing the quality of seeds (Huayta-Hinojosa et al., 2022; Jin et al., 2022; Tetreault et al., 2023). High-vigor seeds are a crucial assurance of successful harvests and higher agricultural product yields since they have apparent growth advantages and output potential (Riveiro et al., 2020). The International Association of Seed Testing (IAST) recommends several methods for determining seed vigor, including germination, cold resistance, accelerated aging, conductivity, and enzyme activity (Fenollosa et al., 2020; Ma et al., 2020; Ali et al., 2022; Zhang et al., 2023). Traditional vigor testing techniques have drawbacks including lengthy measurement times, heavy seed usage, subpar measurement accuracy, and low sensitivity (Peng et al., 2018; Zhu et al., 2019; Pang et al., 2020, Pang et al., 2021). The advancement of seed vigor detection technology has raised the bar for modern agriculture. The hotspot and trend of current mainstream research is machine learning-based detection technology, which is a non-contact direct measuring method with the benefits of being direct, quick, true, and dependable (Medeiros et al., 2020; Wen-ling et al., 2020; Sun et al., 2021; Tu et al., 2023).By using RGB to obtain corn seed images, the authors combined HSI and 3DCNN to establish an optimal classified corn seed vitality model (Fan et al., 2023). In farming, measuring seed vigor is crucial, and a non-destructive machine vision method for detecting seed vigor can aid in a more accurate assessment of seed quality. This provides seed companies with a better basis for decision-making when selecting cultivars and managing plantings (Yasmin et al., 2019; Tu et al., 2023). The digital image of soybean was obtained by using RGB, and the character of soybean was evaluated automatically by using Python Algorithm (Ghimire et al., 2023). The performance of a neural network-based model to identify plant species from paramo seeds via optical RGB images (Ropelewska et al., 2023). High-quality datasets are crucial for accurate machine vision algorithms in seed vigor detection and classification. Yet, current datasets still suffer from several problems below.

(1) Sample imbalance: The dataset for seed vitality has an unequal distribution of seed samples across various categories, leading to a significant gap between the number of samples in each category. It could cause the model to be biased towards predicting categories with more seed samples, thus decreasing the accuracy of predicting categories with fewer samples. Eventually, the accuracy of the model will be negatively impacted.
(2) Sample noise: Obtaining accurate seed vigor sample data is important to ensure proper analysis and model construction. Measurement errors, disturbances during data collection, sampling errors, and other factors can lead to inaccurate data that may mask the true pattern of the data and lead to misinterpretation. Additionally, seeded vitality datasets may contain noisy data, such as mislabeled and duplicate samples, which would negatively impact model training and testing and ultimately reduce model accuracy.
(3) Lack of data diversity: An unbalanced and incomplete distribution of state data in a seeded vitality dataset, even failing to encompass the entire data space or relevant situations, is likely to have a negative impact on the performance of the model and its ability to classify effectively.
(4) Incomplete data: When the data in the seed vitality dataset is incomplete, it means that there are missing values or important features of the seed that are not included, which will affect the accuracy and interpretability of the model.
(5) Inconsistencies in data sources: The dataset contains samples from various seed data sources or collection methods, resulting in differences that cause issues like spatial and temporal inconsistencies between the data. Such inconsistencies would negatively affect the training and prediction of the model and eventually impacts the overall efficacy of the model (Liu et al., 2020).

To improve the accuracy of corn seed vigor detection, a new corn seed vigor dataset was created that included a standard germination test under six contrasting conditions. The dataset includes photographs of corn seeds taken at regular intervals and categorized based on their germination status, primary and secondary root growth. The dataset allows researchers to predicting and grading seed germination and vigor, providing a reliable data source for the study of improving corn seed vigor. Additionally, the method of data collection provides a reference for other seed vigor prediction data collection and improves the validity of non-destructive seed vigor identification and testing data in smart agriculture. It also provides a more scientific approach to seed vigor data collection.

## 2 Values of the data

(1) We conducted an experiment to collect a substantial amount of data on the germination process of maize seeds. The goal was to observe the changes in characteristics, morphology, and color throughout the entire process. Non-fixed shooting was used to capture diverse germination data. By recording germination data at hourly intervals, it was possible to efficiently analyze the seed development process and create accurate and automated testing models for seed quality assessment. This dataset is reliable and useful as it reduces the impact of sample imbalances, inconsistent data sources, and incomplete data.

(2) Seed vigor detection greatly benefits from the seed dataset of RGB images, which is enriched in feature information including morphology, structure, and texture. Compared to traditional methods, RGB images of seeds offer advantages such as low cost, easy obtainability, non-contact capability, and low computational consumption. These advantages make nondestructive seed vigor detection more practical and valuable for research and application.

(3) The datasets complement databases for the detection of seed vigor and standard germination processes. These data are vital for researching seed vigor classification, predicting germination, and evaluating and detecting vigor. By analyzing the data, the researchers can accurately detect seed vigor and predict germination ability, leading to improved seed quality and crop yield.

## 3 Materials and methods

### 3.1 Selection of materials

**Figure 1:**
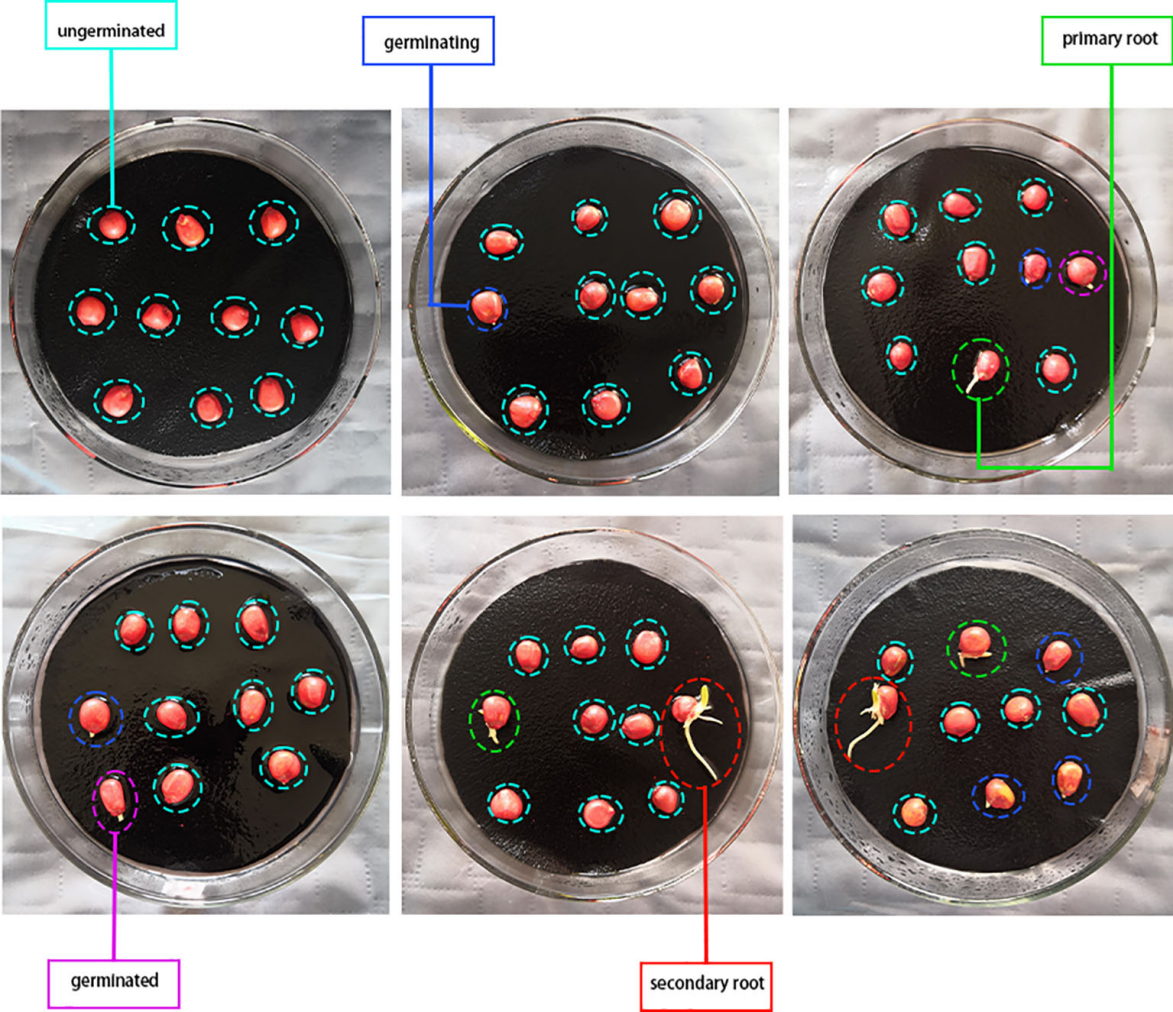
Different germination states of maize seeds in standard germination test.

In the experiment, we selected Meiyu 817 maize seeds. The seeds are known for their strong resistance, high production rate, and wide cultivation in Northeast China. **Figure 1** displays the various stages of germination for the sample, including ungerminated, germinating, germinated, primary root, and secondary root. These stages depict the different phases of seed germination, and the sample is visible in **Figure 1** below.

### 3.2 Experimental condition

The experiment consisted of six groups, with each group consisting of 200 seeds. Initially, the maize seeds were categorized into three groups based on the aging experiment, with aging times of 0d, 3d, and 6d, respectively, in a 45°C constant temperature oven. The remaining three groups were stored in environments with temperatures of 20°C, 0°C, and -20°C, respectively. The seed grouping and 100-grain weight are presented in **Table 1** below.

**Table 1:** Experimental groupings.

Experiment number	Number of grains (pcs)	Prerequisite	Hundred grains weight (g)
1	200	20°C	36.68
2	200	0°C	37.29
3	200	-20°C	37.04-37.05
4	200	age 3d; 45°C	36.93-36.94
5	200	age 6d; 45°C	36.5
6	200	age 9d; 45°C	36.57

### 3.3 Standard germination test

According to the Technical Regulations on Crop Seed Germination (GB/T 3543.4-1995), the seeds were placed in a germination chamber that maintained a constant temperature of 25°C. There were six subgroups, each consisting of 200 seeds, with varying temperatures (-20°C, 0°C, 20°C) and durations (3d, 6d, 9d). Then, placed 20 subgroups of each subgroup in Petri dishes, for a total of 120 Petri dishes with 10 seeds each (1,200 seeds in total). After that, the seeds were evenly spaced to ensure enough space for growth and to prevent mold. Finally, sprayed water every 2-3 hours to keep the bed moist, and did not cover the dishes to ensure sufficient oxygen supply. As for data collection, diffused light was used during the day and 45w incandescent light at night to photograph the germination process. A Huawei Honor V10 mobile phone was used to take photos from a height of 20-25cm every hour. However, we removed any moldy or dead seeds and labeled the remaining seeds into five categories based on their germination vigor. 1. ungerminated; 2. germinating; 3. germinated; 4. primary root; 5. secondary root.

**Figure 2:**
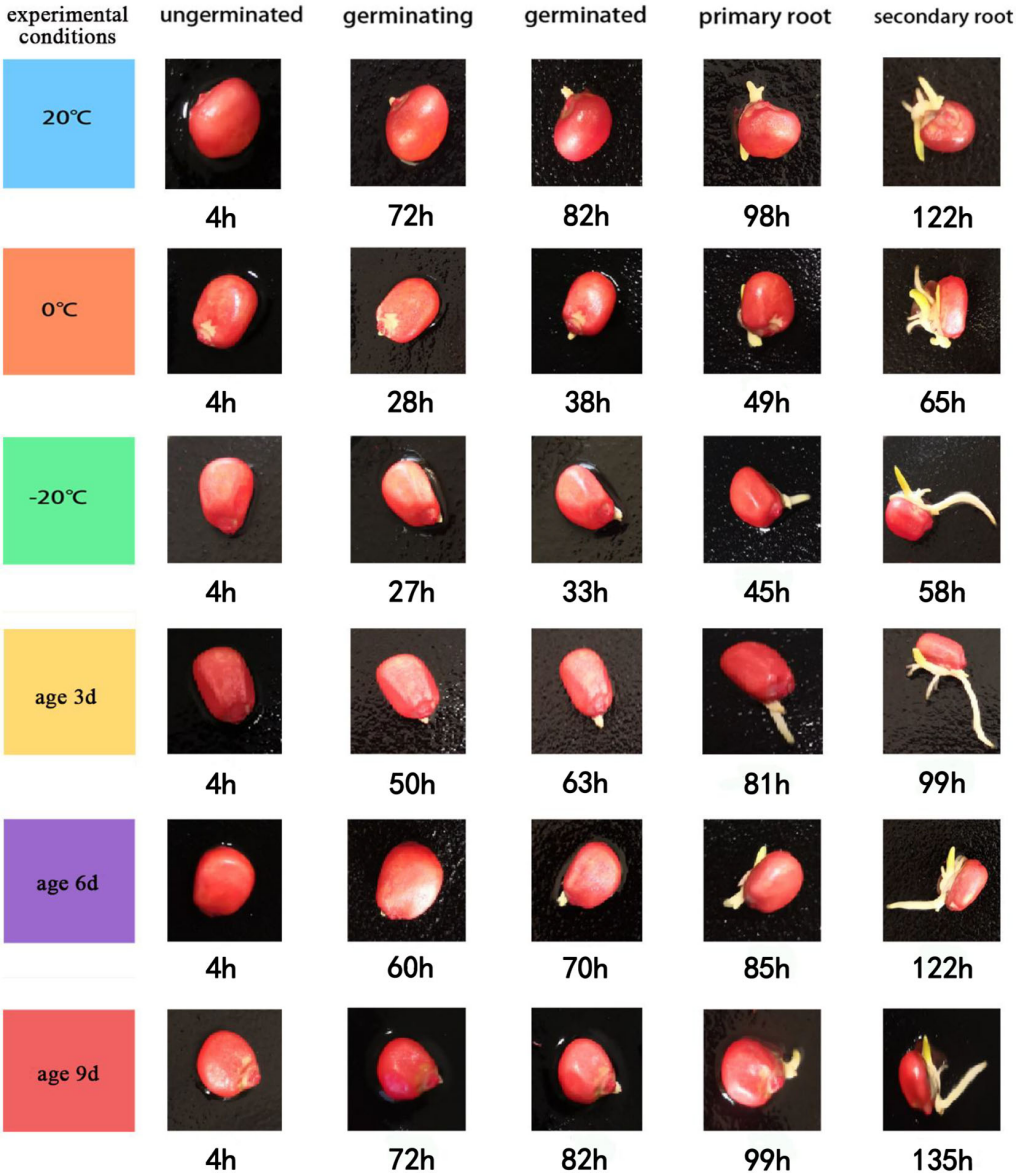
Chronological germination status of six seed groups.

The corresponding germination states were: (1) seeds were not germinated, decayed, or dead; (2) primary root 0-2mm; (3) primary root germinated 2mm; (4) there was and there was only one primary root, and the seed primary root exceeded more than 2mm; (5) there was more than one secondary root in addition to the primary root. **Figure 2** below shows the time series of seed germination status of 6 groups of comparison tests:

### 3.4 Construction of the dataset

During the germination tests, photos were taken for a total of 6 days and 21 hours. The test was concluded 3 hours earlier than the specified 7-day time periods, as the samples had already reached their highest germination rate. One photo was taken per hour throughout the experiment, and the result was 120 photographs per hour. Throughout the experiment, one photo was taken every hour, resulting in 120 photos per hour. In total, 19,800 RGB images with 3456 x 4608 pixels, were collected and annotated using LabelImg. Data annotation is stored in the format of the PASCAL VOC dataset, and is set to.xml format. A total of 181,250 valid data were labeled, while fuzzy or obscured images were removed to reduce data noise. The number of labels corresponding to the five different states were: 1. ungerminated:149842; 2. germinating:7042; 3. germinated:1936; 4. primary root:5087; 5. secondary root:17343. For easier download, we uploaded the 120-folder dataset separately, which was generated each hour. It could be accessed on the Kaggle public dataset titled Seed Vigor Detection RGB Image. The dataset is available at the following two address: https://www.kaggle.com/datasets/chengchengchen/seed-vigor-detection-rgb-image http://ieee-dataport.org/documents/rgb-image-dataset-seed-germination-prediction-and-seed-vigor

### 3.5 Seed viability object detection experiments

In order to verify the validity of the dataset, we perform experiments on the seeds vitality object detection using the two-stage object detection model Faster RCNN (Girshick, 2015), the one-stage model SSD (Liu et al., 2016), YOLOv3 (Redmon and Farhadi, 2018), YOLOv5 (Jocher et al., 2020), RTMDet (Lyu et al., 2022), and the anchor-free model FCOS (Tian et al., 2019); and we optimize the feature extraction capability by change the backbone. The average accuracy mAP of several target categories, the recognition accuracy mAP50 and mAP75 for IoU thresholds of 0.5 and 0.75, and the recognition accuracy of each category are recorded in the experiment results.

All experiments are run on a server with an Inter(R) Xeon(R) Platinum 8336C CPU at 2.3 GHz, two NVIDIA GeForce RTX 3090 24G GPUs, and 256 GB of RAM. The experimental results are shown in **Table 2**, and it could be seen that the results of two-stage network structure detection are better than the one-stage and anchor-free models, where backbone is ResNeXt101, the network combined with FPN and Faster RCNN provides the best results. The recognition accuracy of RTMDet in one-stage is the highest. The recognition accuracies of the one-stage model and the anchor free model are lower in the categories Germinating and Germinated, but the two-stage model significantly improves the recognition accuracies of these two categories. It indicates that the average recognition accuracy as well as the recognition accuracy of a single category can be changed by adjusting the backbone, adding FPN structure, and adjusting the complexity of the model structure.

**Table 2:** Seed Viability Object Detection Experiments.

Model	Backbone	mAP (%)	mAP .5(%)	mAP .75(%)	Ungerminated	Germinating	Germinated	Primary root	Secondary root
Faster RCNN	ResNet50	0.765	0.908	0.896	0.836	0.696	0.617	0.799	0.875
Faster+FPN	ResNet 50	0.783	0.921	0.912	0.854	0.734	0.640	0.815	0.873
Faster+FPN	ResNeXt101	0.808	0.943	0.931	0.862	0.758	0.705	0.83	0.887
YOLOV3-SPP	Darknet53	0.635	0.826	0.792	0.813	0.563	0.391	0.695	0.711
FCOS	ResNet50	0.637	0.796	0.763	0.839	0.566	0.379	0.703	0.699
SSD	VGG	0.512	0.689	0.625	0.779	0.168	0.318	0.633	0.664
RTMDet	CSPDarknet	0.757	0.887	0.87	0.85	0.682	0.562	0.801	0.889
YOLOv5-n	CSPDarknet	0.625	0.758	0.739	0.842	0.461	0.3	0.729	0.795
YOLOv5-s	CSPDarknet	0.739	0.868	0.855	0.863	0.709	0.47	0.8	0.851

## Data availability statement

The datasets presented in this study can be found in online repositories. The names of the repository/repositories and accession number(s) can be found in the article/supplementary material.

## Author contributions

CC: Conceptualization, Formal analysis, Funding acquisition, Investigation, Methodology, Resources, Software, Supervision, Validation, Visualization, Writing – original draft, Writing – review & editing. MB: Data curation, Formal analysis, Visualization, Writing – original draft. TW: Data curation, Formal analysis, Validation, Visualization, Writing – original draft. WZ: Data curation, Formal analysis, Visualization, Writing – original draft. HY: Conceptualization, Funding acquisition, Methodology, Project administration, Resources, Supervision, Writing – review & editing. TP: Conceptualization, Formal analysis, Writing – review & editing. JW: Data curation, Funding acquisition, Writing – original draft. ZL: Conceptualization, Funding acquisition, Investigation, Writing – original draft. XW: Funding acquisition, Supervision, Writing – original draft.

## References

[B1] Ali F. Qanmber G. Li F. Wang Z. (2022). Updated role of ABA in seed maturation, dormancy, and germination. J. Adv. Res. 35, 199–214. doi: 10.1016/j.jare.2021.03.011 35003801 PMC8721241

[B2] Collins W. Kandpal L. M. Lee H. Bae H. (2018). Rapid assessment of corn seed viability using short wave infrared line-scan hyperspectral imaging and chemometrics. Sensors Actuators B Chem. 255, 498–507. doi: 10.1016/j.snb.2017.08.036

[B3] Fan Y. An T. Wang Q. Yang G. Huang W. Wang Z. . (2023). Non-destructive detection of single-seed viability in maize using hyperspectral imaging technology and multi-scale 3D convolutional neural network. Front. Plant Sci. 14. doi: 10.3389/fpls.2023.1248598 PMC10497746 37711294

[B4] Fenollosa E. Jene L. Munne-Bosch S. (2020). A rapid and sensitive method to assess seed longevity through accelerated aging in an invasive plant species. Plant Methods 16, 64. doi: 10.1186/s13007-020-00607-3 32411273 PMC7206761

[B5] Ghimire A. Kim S. H. Cho A. Jang N. Ahn S. Islam M. S. . (2023). Automatic Evaluation of Soybean Seed Traits Using RGB Image Data and a Python Algorithm. Plants (Basel) 12, 3078–3088. doi: 10.3390/plants12173078 37687325 PMC10490075

[B6] Girshick R. (2015). “Fast r-cnn,” in Proceedings of the IEEE international conference on computer vision in 2015 IEEE International Conference on Computer Vision (ICCV), Vol. 12 (Santiago, Chile: IEEE), 1440–1448. doi: 10.1109/ICCV.2015.169

[B7] Hall R. M. Urban B. Skalova H. Karrer G. (2021). Seed viability of common ragweed (Ambrosia artemisiifolia L.) is affected by seed origin and age, but also by testing method and laboratory. NEOBIOTA 70, 193–221. doi: 10.3897/neobiota:70.66915

[B8] Huayta-Hinojosa L. D. Nolasco-Lozano E. Guerra D. Hermoza-Gamboa J. Quispe-Melgar H. R. (2022). Performance and physiological quality of Escallonia resinosa seeds: prospects for their use in reforestation and restoration. Restor. Ecol. 31, 1–12. doi: 10.1111/rec.13848

[B9] Jin B. Qi H. Jia L. Tang Q. Gao L. Li Z. . (2022). Determination of viability and vigor of naturally-aged rice seeds using hyperspectral imaging with machine learning. Infrared Phys. Technol. 122, 104097. doi: 10.1016/j.infrared.2022.104097

[B10] Jocher G. Stoken A. Borovec J. Changyu L. Hogan A. Diaconu L. . (2020). ultralytics/yolov5: v3. 0. Zenodo 8, 1–6. doi: 10.5281/ZENODO.3983579

[B11] Liu W. Anguelov D. Erhan D. Szegedy C. Reed S. Fu C. Y. . (2016). “Ssd: Single shot multibox detector,” in *Lecture Notes in Computer Science* . (Amsterdam, NETHERLANDS: SPRINGER-VERLAG BERLIN). 21–37. doi: 10.1007/978-3-319-46448-0_2

[B12] Liu U. Cossu T. A. Davies R. M. Forest F. Dickie J. B. Breman E. (2020). Conserving orthodox seeds of globally threatened plants ex situ in the Millennium Seed Bank, Royal Botanic Gardens, Kew, UK: the status of seed collections. Biodiversity Conserv. 29, 2901–2949. doi: 10.1007/s10531-020-02005-6

[B13] Lyu C. Zhang W. Huang H. Zhou Y. Wang Y. Liu Y. . (2022). Rtmdet: An empirical study of designing real-time object detectors. arXiv preprint arXiv 4, 1–15. doi: 10.48550/arXiv.2212.07784

[B14] Ma T. Tsuchikawa S. Inagaki T. (2020). Rapid and non-destructive seed viability prediction using near-infrared hyperspectral imaging coupled with a deep learning approach. Comput. Electron. Agric. 177, 105683. doi: 10.1016/j.compag.2020.105683

[B15] Medeiros A. Pinheiro D. T. Xavier W. A. Silva L. Dias D.C.F.d. S. (2020). Quality classification of Jatropha curcas seeds using radiographic images and machine learning. Ind. Crops Products 146, 112162. doi: 10.1016/j.indcrop.2020.112162

[B16] Pang L. Wang J. Men S. Yan L. Xiao J. (2021). Hyperspectral imaging coupled with multivariate methods for seed vitality estimation and forecast for Quercus variabilis. Spectrochim Acta A Mol. Biomol Spectrosc 245, 118888. doi: 10.1016/j.saa.2020.118888 32947159

[B17] Pang L. Xiao J. Ma J. Yan L. (2020). Hyperspectral imaging technology to detect the vigor of thermal-damaged Quercus variabilis seeds. J. Forestry Res. 32, 461–469. doi: 10.1007/s11676-020-01144-4

[B18] Peng Y. Zhao F. Bai J. Zheng X. Wang W. Sun Q. (2018). Tomato seed vigor detection and grading based on mapping features. Transact. Chinese Soc. Agric. Machinery 49, 327–333. doi: 10.6041/j.issn.1000-1298.2018.02.042

[B19] Redmon J. Farhadi A. (2018). Yolov3: An incremental improvement. arXiv preprint arXiv 8, 1–6. doi: 10.48550/arXiv.1804.02767

[B20] Riveiro S. F. Cruz Ó. Casal M. Reyes O. (2020). Fire and seed maturity drive the viability, dormancy, and germination of two invasive species: Acacia longifolia (Andrews) Willd. and Acacia mearnsii De Wild. Ann. For. Sci. 77, 60–70. doi: 10.1007/s13595-020-00965-x

[B21] Ropelewska E. Kruczyńska D. E. Mieszczakowska-Frąc M. (2023). Distinguishing Seed Cultivars of Quince (Cydonia oblonga Mill.) Using Models Based on Image Textures Built Using Traditional Machine Learning Algorithms. Agriculture 13, 1310. doi: 10.3390/agriculture13071310

[B22] Song P. Yue X. Gu Y. Yang T. (2022). Assessment of maize seed vigor under saline-alkali and drought stress based on low field nuclear magnetic resonance. Biosyst. Eng. , 220, 135–145. doi: 10.1016/j.biosystemseng.2022.05.018

[B23] Sun J. Zhang L. Zhou X. Wu X. Shen J. Dai C . (2021). Detection of rice seed vigor class using hyperspectral image depth features. Transact. Chinese Soc. Agric. Engineering 37, 171–178. doi: 10.11975/j.issn.1002-6819.2021.14.019

[B24] Tetreault H. Fleming M. Hill L. Dorr E. Yeater K. Richards C. . (2023). A power analysis for detecting aging of dry-stored soybean seeds: Germination versus RNA integrity assessments. Crop Sci. 63, 1481–1493. doi: 10.1002/csc2.20821

[B25] Tian Z. Shen C. Chen H. He T. (2019). “Fcos: Fully convolutional one-stage object detection,” in Proceedings of the IEEE/CVF international conference on computer vision. (Seoul, Korea (South): IEEE), 9627–9636. doi: 10.1109/ICCV.2019.00972

[B26] Tu K. Cheng Y. Ning C. Yang C. Dong X. Cao H. . (2022). Non-Destructive Viability Discrimination for Individual Scutellaria baicalensis Seeds Based on High-Throughput Phenotyping and Machine Learning. Agriculture 12, 1616. doi: 10.3390/agriculture12101616

[B27] Tu K. Wu W. Cheng Y. Zhang H. Xu Y. Dong X. . (2023). AIseed: An automated image analysis software for high-throughput phenotyping and quality non-destructive testing of individual plant seeds. Comput. Electron. Agric. 207, 107740. doi: 10.1016/j.compag.2023.107740

[B28] Wen-ling J. I. N. Nai-liang C. A. O. Ming-dong Z. H. U. Wei C. Pei-guang Z. Qing-lei Z. . (2020). Nondestructive grading test of rice seed activity using near infrared super-continuum laser spectrum. Chin. Optics 13, 1032–1043. doi: 10.37188/co.2020-0027

[B29] Xanthopoulos G. (2020). Viability modelling of seeds and sensitivity analysis under fluctuating temperature and moisture content. J. Stored Products Res. 89, 101708. doi: 10.1016/j.jspr.2020.101708

[B30] Yasmin J. Raju Ahmed M. Lohumi S. Wakholi C. Kim M. S. Cho B. K. (2019). Classification Method for Viability Screening of Naturally Aged Watermelon Seeds Using FT-NIR Spectroscopy. Sensors (Basel) 19, 1190–1204. doi: 10.3390/s19051190 30857184 PMC6427422

[B31] Zhang Y. Song X. Zhang W. Liu F. Wang C. Liu Y. . (2023). Maize PIMT2 repairs damaged 3-METHYLCROTONYL COA CARBOXYLASE in mitochondria, affecting seed vigor. Plant J. 115, 220–235. doi: 10.1111/tpj.16225 36999611

[B32] Zhu S. Zhang J. Chao M. Xu X. Song P. Zhang J. . (2019). A Rapid and Highly Efficient Method for the Identification of Soybean Seed Varieties: Hyperspectral Images Combined with Transfer Learning. Molecules 25, 152–166. doi: 10.3390/molecules25010152 31905957 PMC6982693

